# A Rapidly Progressive Thrombosed Middle Cerebral Artery Aneurysm After Endovascular Treatment

**DOI:** 10.7759/cureus.104794

**Published:** 2026-03-06

**Authors:** Ryuji Matsushita, Kouhei Nii, Ritsurou Inoue, Naoki Wakuta, Hiroshi Abe

**Affiliations:** 1 Neurosurgery, Fukuoka University Chikushi Hospital, Chikushino, JPN; 2 Stroke Center, Fukuoka University Chikushi Hospital, Chikushino, JPN; 3 Neurosurgery, Fukuoka University Hospital, Fukuoka, JPN

**Keywords:** coil embolization, intracranial ruptured aneurysm, middle cerebral artery, recurrence, thrombus formation

## Abstract

Endovascular coil embolization for ruptured cerebral aneurysms generally yields favorable outcomes; however, concerns regarding long-term recurrence persist. We report a rare case of a ruptured middle cerebral artery aneurysm treated with coil embolization that exhibited rapid enlargement over several months, accompanied by progressive thrombus formation. The clinical course and imaging findings are presented together with a review of the relevant literature. A 32-year-old woman presented with a sudden-onset headache and was diagnosed with subarachnoid hemorrhage. Angiography revealed a 2-mm saccular aneurysm in the right middle cerebral artery, for which endovascular coil embolization was performed. The postoperative course was uneventful. Follow-up angiography performed 13 days after treatment demonstrated only a small residual neck, and outpatient surveillance was planned. Although her clinical status remained stable, follow-up magnetic resonance imaging obtained four months later revealed recurrence of the aneurysm, which had enlarged to 10 mm with partial intraluminal thrombosis and associated perianeurysmal edema. Given the rapid enlargement, urgent preoperative cerebral angiography was performed, followed by surgical clipping. Subsequent follow-up demonstrated no further recurrence. Several factors have been identified as risks for recurrence after endovascular coiling of cerebral aneurysms, including large aneurysm size, low coil packing density, and a history of rupture. As illustrated by the present case, even aneurysms that are initially small may undergo substantial enlargement over a short period when associated with intraluminal thrombosis. These findings underscore the necessity of rigorous postoperative imaging surveillance.

## Introduction

Endovascular coil embolization for ruptured cerebral aneurysms is widely accepted as an acute treatment option [1]. This procedure prevents subsequent aneurysm rupture by filling the aneurysmal sac with platinum coils via a microcatheter, thereby inducing blood flow stasis. However, concerns remain regarding its higher long-term recurrence rate compared with microsurgical clipping [2]. Aneurysm recurrence can result in catastrophic intracranial hemorrhage and severe neurologic deficits [3]. Large or inadequately coil-packed aneurysms are highly susceptible to recurrence driven by coil compaction [4,5], whereas small aneurysms generally show a lower incidence of short-term recurrence due to minimal residual blood flow relative to packing volume. In some instances, large aneurysms disguised as small lesions due to partial thrombosis may manifest an aggressive growth pattern in the short term [6]. Other potential contributing factors may include hemodynamic stress and inflammatory changes [7]. We describe a rare case in which a very small ruptured middle cerebral artery (MCA) aneurysm exhibited rapid post-treatment enlargement with progressive thrombus formation over several months following coil embolization. The clinical course is presented together with a literature-based discussion of the potential mechanisms underlying aneurysm enlargement.

## Case presentation

**Figure 1:**
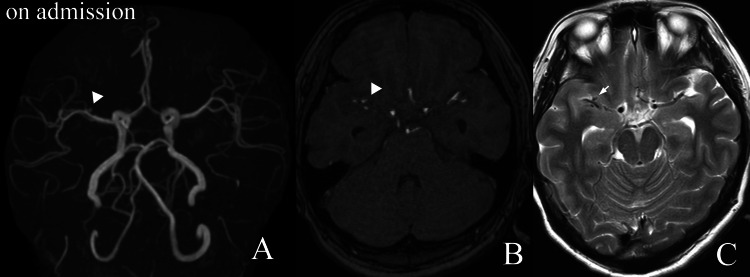
Magnetic resonance imaging findings at admission. (A) Time-of-flight magnetic resonance angiography (TOF-MRA) and (B) contrast-enhanced magnetic resonance angiography demonstrate a 2-mm saccular aneurysm arising from the M2 superior trunk of the right middle cerebral artery (arrowhead). (C) T2-weighted imaging shows no perianeurysmal signal change suggestive of thrombus formation (arrow).

A 32-year-old woman was emergently transported to our hospital with sudden-onset headache and impaired consciousness. On arrival, her level of consciousness was Japan Coma Scale II-30, and headache was the only focal neurological finding (Hunt and Kosnik grade 3). Head computed tomography revealed diffuse subarachnoid hemorrhage predominantly around the right Sylvian fissure, and magnetic resonance angiography demonstrated a saccular aneurysm of the right MCA (Figure 1).

**Figure 2:**
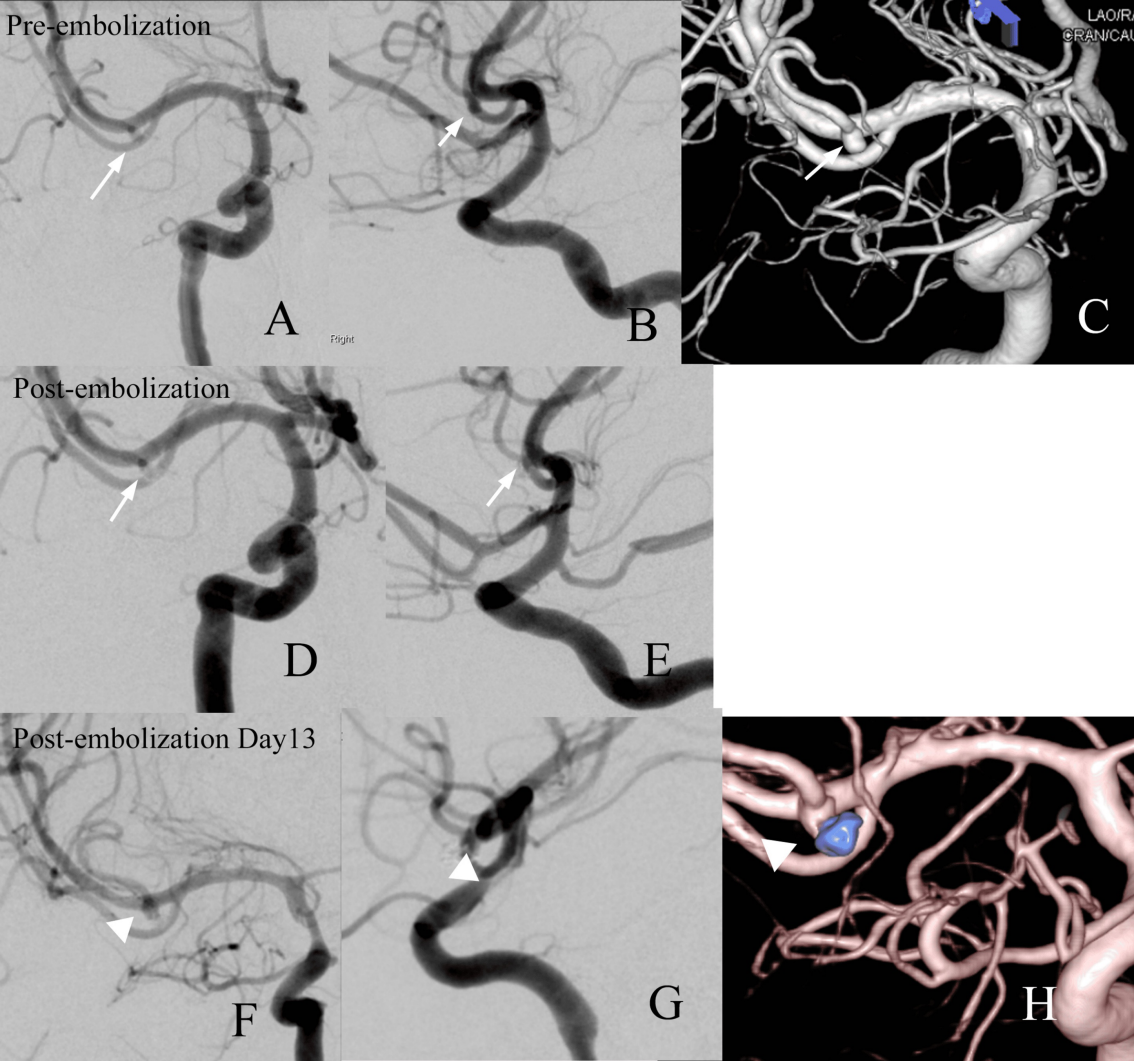
Digital Subtraction Angiography (DSA) findings. (A) Anteroposterior and (B) lateral DSA views and (C) three-dimensional (3D) DSA obtained before treatment demonstrate a downward-projecting aneurysm arising from the distal portion of the M2 superior trunk of the right middle cerebral artery, measuring 2 mm in dome diameter with a 1-mm neck (arrow). (D) Post-treatment anteroposterior and (E) lateral DSA views show aneurysm occlusion following placement of a single 2-mm coil, with a small neck remnant (arrow). (F) Anteroposterior, (G) lateral, and (H) 3D DSA obtained on post-coiling Day 13 demonstrate enlargement of the neck remnant due to coil compaction. Intra-aneurysmal flow remained absent, and conservative follow-up was selected (arrowhead).

To prevent re-rupture, endovascular coil embolization was scheduled for the same day. Under general anesthesia, an 8-Fr guiding catheter was advanced from the femoral artery to the right internal carotid artery (ICA). Right internal carotid angiography confirmed a small saccular aneurysm (dome diameter 2 mm, neck diameter 1 mm) at the M2 bifurcation of the MCA. A microcatheter was navigated into the aneurysm, and occlusion was achieved using a single 2-mm coil, resulting in complete cessation of intra-aneurysmal flow (Figure 2).

**Figure 3:**
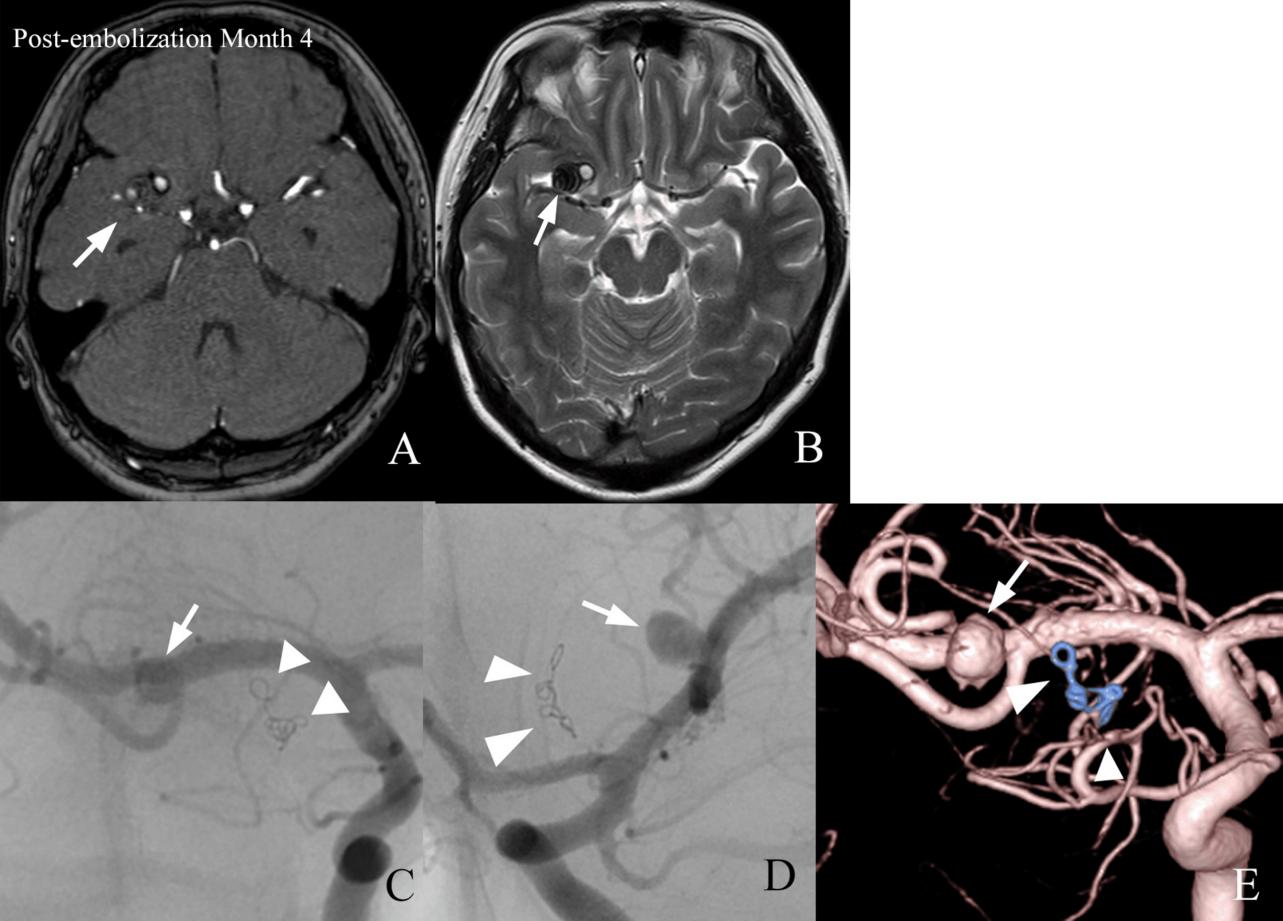
Follow-up imaging at four months after treatment (A) Time-of-flight magnetic resonance angiography and (B) T2-weighted imaging demonstrate an enlarged aneurysm measuring 12 mm in diameter, containing mixed high- and low-signal intensities suggestive of intraluminal thrombus (arrow). (C) Anteroposterior, (D) lateral, and (E) three-dimensional (3D) digital subtraction angiography (DSA) demonstrate further aneurysm enlargement, measuring 14 mm in dome diameter with a 4-mm neck (arrow). The coil mass is displaced anteriorly toward the non-opacified thrombosed portion of the aneurysm (arrowhead).

The patient’s level of consciousness gradually improved, and the intracranial hematoma resolved. No new neurological deficits developed, and no perioperative complications were observed. Follow-up angiography performed on day 13 demonstrated only a minimal neck remnant of the MCA aneurysm; however, because further endovascular treatment was considered technically challenging, outpatient imaging surveillance was planned. The patient was completely asymptomatic with no focal neurological deficits after discharge. However, a routine follow-up MRI performed four months after treatment revealed a thrombosed aneurysm measuring 12 mm with surrounding edema (Figure 3A, 3B). Despite the absence of clinical symptoms, the patient was hospitalized for further evaluation because of the high risk of re-rupture associated with recurrent aneurysms. Subsequent cerebral angiography demonstrated displacement of the coil mass from the middle cerebral artery and an enlarged aneurysm (dome diameter 14 mm, neck diameter 4 mm) (Figure 3C).

**Figure 4:**
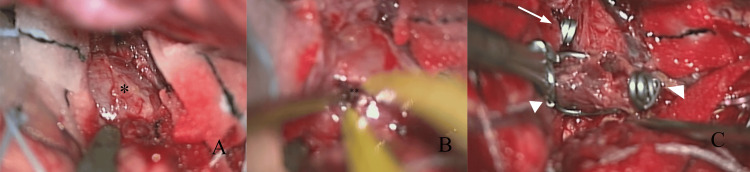
Intraoperative image (A) After microsurgical dissection of the Sylvian fissure, the aneurysm was identified proximal to the M2 superior trunk of the middle cerebral artery (asterisk). (B) The aneurysm sac was opened, and the intraluminal thrombus (double asterisk) was removed to achieve decompression. (C) Following reduction of the aneurysm sac, definitive clipping of the aneurysm neck was performed using an L-shaped clip (arrow) and a temporal clip (arrowhead).

Craniotomy and surgical clipping were planned to prevent re-rupture. The patient underwent a right frontotemporal craniotomy under general anesthesia five months after the initial endovascular treatment. Using a transsylvian approach, the aneurysm was identified at the proximal portion of the M2 superior trunk. Temporary clips were applied to the proximal ICA and distal MCA to achieve proximal and distal control. The aneurysm sac was then opened, and the intraluminal thrombus was removed. Multiple clips were subsequently applied across the aneurysm neck to achieve complete obliteration (Figure 4).

**Figure 5:**
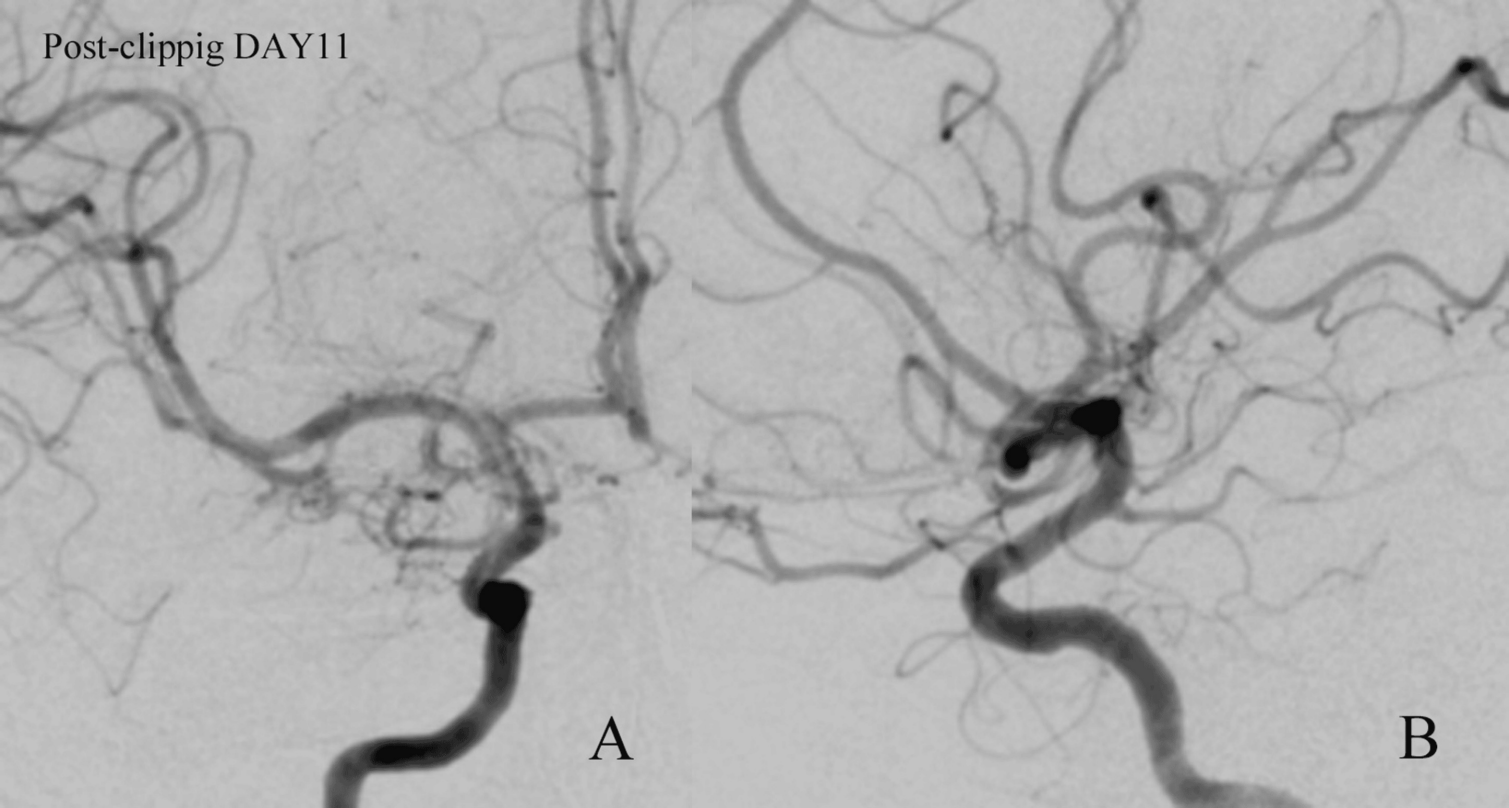
Digital Subtraction Angiography after treatment (A) Anteroposterior and (B) lateral digital subtraction angiography demonstrated no evidence of aneurysm recurrence.

No new neurological deficits were observed postoperatively, and angiography performed on postoperative day 11 demonstrated complete obliteration of the aneurysm (Figure 5).

Follow-up MRI obtained after discharge revealed no new intracranial abnormalities, and cerebral angiography performed four months after clipping confirmed the absence of aneurysm recurrence.

## Discussion

In the present case, the aneurysm measured only 2 mm at the time of initial treatment, and MRI showed no findings suggestive of a rapidly enlarging thrombosed aneurysm. Although aneurysm size - typically the strongest predictor of recurrence after coiling - was not a concern, the patient exhibited several established risk factors, including a history of rupture, a small neck remnant, and aneurysm location within the MCA territory [4,5]. While coil compaction is the most common cause of post-embolization recurrence, the rapid enlargement observed in this case suggests that mechanisms beyond coil compaction - particularly hemodynamic and inflammatory factors - likely contributed to aneurysm progression. Although vascular dissection was considered as a potential contributing mechanism, careful review of preoperative imaging and intraoperative findings revealed no evidence of arterial dissection, such as an intimal flap, double lumen, or vessel wall irregularity. Therefore, vascular dissection was considered unlikely to have contributed to aneurysm progression in this case.

In recurrent aneurysms after endovascular treatment, high wall shear stress (WSS) at the aneurysm neck has been shown to induce endothelial injury and promote turbulent flow, thereby triggering recurrence [7]. Such localized hemodynamic stress drives chronic inflammation through endothelial activation and cytokine production, ultimately leading to pathological remodeling of the aneurysm wall.

In the present case, chronic inflammatory responses dominated by macrophages were considered central to the progression of the thrombosed aneurysm. Macrophage accumulation within the aneurysm wall promotes cytokine release, smooth muscle cell apoptosis, and progressive weakening of the structural integrity of the wall [6]. Furthermore, neovascularization of the vasa vasorum has been reported to contribute to wall fragility and aneurysm enlargement [6], supporting the idea that inflammation-driven vascular remodeling underlies aneurysm growth and recurrence. Chronic inflammation induces vasa vasorum proliferation, which enhances oxygen delivery but also creates entry routes for inflammatory cell infiltration. These newly formed microvessels are structurally fragile and highly permeable, and repeated intramural hemorrhages worsen wall degeneration, promote mural thickening, and stimulate additional thrombus formation [8]. This cycle of inflammation, neovascularization, intramural hemorrhage, and recurrent inflammation is believed to accelerate the progression of thrombosed aneurysms. In this case, contrast-enhanced MRI was not performed because of early surgical intervention; however, the presence of perianeurysmal edema was considered indicative of chronic inflammatory activity within the aneurysm wall.

Additional treatment options for recurrent aneurysms after coil embolization can be broadly categorized into further endovascular interventions and microsurgical approaches. Endovascular strategies, including stent-assisted coil embolization and flow diverter placement, have demonstrated favorable outcomes in selected cases [9,10]. Microsurgical clipping offers the advantage of achieving definitive occlusion even in recurrences following endovascular treatment [11]. In certain situations, parent vessel occlusion combined with distal bypass may also be considered [12]. In the present case, the rapidly enlarging thrombosed aneurysm required urgent retreatment, particularly given the patient's history of rupture. Additional endovascular options posed several concerns: (i) the need for perioperative dual antiplatelet therapy, which requires time to become effective, and (ii) an increased risk of intraoperative and postoperative bleeding. Moreover, repeat endovascular intervention raised the possibility of further recurrence and was unlikely to contribute to the resolution of the surrounding cerebral edema. Given these considerations, microsurgical clipping was selected, resulting in successful cessation of aneurysm growth and improvement of perianeurysmal edema.

## Conclusions

We report a case of a ruptured MCA aneurysm that exhibited rapid post-treatment enlargement with progressive thrombus formation shortly after endovascular coil embolization. Even small saccular aneurysms may undergo thrombus organization driven by chronic inflammation, recurrent neovascularization, and intramural hemorrhage, highlighting the importance of rigorous imaging surveillance. Rapidly enlarging thrombosed aneurysms warrant prompt consideration of microsurgical intervention to prevent re-rupture or deterioration of neurological function due to mass effect.
